# Genome-wide identification of the *Glycine max* (soybean) aspartate kinase (AK) gene family and functional analysis of the *GmAK6* gene in seed amino acid metabolism

**DOI:** 10.3389/fpls.2026.1868742

**Published:** 2026-08-04

**Authors:** Chao Fan, Wenwei Liang, Wei Li, Jianxin Liu, Miao Liu, Guang Yang, Shufeng Di, Heyi Ma, Menglong Chai, Lili Tang, Yingdong Bi

**Affiliations:** 1 Institute of Crop Cultivation and Tillage, Heilongjiang Academy of Agricultural Sciences, Harbin, China; 2 College of Agriculture, Northeast Agricultural University, Harbin, China; 3 Institute of Industrial Crops, Heilongjiang Academy of Agricultural Sciences, Harbin, China

**Keywords:** amino acid metabolism, aspartate kinase gene family, bioinformatics analysis, gene expression profiling, *Glycine max*

## Abstract

**Introduction:**

Aspartate kinase (AK) functions as a key regulatory enzyme in seed development and amino acid metabolism in plants. Although soybean (*Glycine max*) is a model high protein crop rich in essential amino acids, systematic characterization of its AK gene family remains limited.

**Methods:**

To identify AK family genes at the genome wide level and analyze their functions, we performed a comprehensive bioinformatic analysis of the soybean genome, including phylogenetic reconstruction, promoter cis element prediction, transcriptomic profiling, and subcellular localization via GFP fusion assays. Functional validation was conducted using *GmAK6* overexpressing transgenic soybean lines, with amino acid profiles quantified by HPLC.

**Results:**

A total of 16 *GmAK* genes were identified, unevenly distributed across 10 chromosomes, with segmental duplication as the primary evolutionary driver of family expansion. Promoter analysis revealed that nearly half of the cis acting elements are associated with phytohormone signaling (e.g., jasmonic acid, abscisic acid) and abiotic stress responses (e.g., wounding, low temperature). Transcriptomic data showed distinct spatiotemporal expression patterns, with *GmAK5* and *GmAK6* significantly upregulated during seed development. GmAK6 was localized to chloroplasts. Amino acid profiling of *GmAK6* overexpressing seeds demonstrated increased accumulation of methionine, threonine, and cysteine, alongside reduced levels of glutamate and proline.

**Discussion:**

These results suggest that *GmAK6* plays a regulatory role in amino acid metabolism during seed development and highlight the functional importance of AK genes in soybean.

## Introduction

Soybean (*Glycine max*) is an essential agricultural crop that serves as a principal source of plant-derived protein and edible oil worldwide (Yang et al., 2022). It is recognized as a plant-based source of high-quality protein, providing all essential amino acids required for human nutrition (Aschemann-Witzel et al., 2021). The increasing demand for soybean-derived protein has reinforced its dominance in the global plant protein market, where it accounts for more than 50% of total production (Messina, 2022). Soybean seeds contain approximately 40% protein, and their nutritional quality is comparable to that of egg protein, which is widely regarded as a reference standard for amino acid evaluation (Kudełka and Kowalska, 2021). The amino acid composition of soybean protein closely resembles that of animal protein, particularly with respect to essential amino acids (Kim and Krishnan, 2004; Liu et al., 2023). This nutritional profile underscores the importance of soybeans in improving human nutrition and supporting global food security (Wang et al., 2018).

Amino acid metabolism in plants is tightly regulated during seed development, where seeds function as major sites of storage and metabolic activity (Dong et al., 2024). Seed development is governed by a complex metabolic network in which aspartate-derived amino acids, including lysine (Lys), methionine (Met), threonine (Thr), and isoleucine (Ile), occupy central regulatory positions (Duo et al., 2024; Han et al., 2021). The initial step in this biosynthetic pathway involves the phosphorylation of aspartate to aspartate-4-phosphate, catalyzed by monofunctional aspartate kinases (AKs) and bifunctional aspartate kinase-homoserine dehydrogenase (AK-HSDH) (Jander and Joshi, 2010). Aspartyl-4-phosphate is subsequently converted into aspartate semialdehyde, which serves as a critical branch-point intermediate for the biosynthesis of lysine, methionine, threonine, and isoleucine. Both AKs and AK-HSDH enzymes belong to the AK gene family (EC 2.7.2.4) (Han et al., 2018; Yang et al., 2011). Enzymatic activity within this pathway is subject to feedback inhibition mediated by lysine and threonine, and this coordinated regulation directly influences downstream biosynthesis of Met and Ile (Petit et al., 2018). In *Arabidopsis thaliana*, three AK genes (*AK1* [*At5g13280*], *AK2* [*A5g14060*], and *AK3* [*At3g02020*]) and two AK-HSDH genes (*AK-HSDH1* [*At1g31230*] and *AK-HSDH2* [*At4g19710*]) have been identified (Jander and Joshi, 2010), and expression profiling indicates that these five genes are ubiquitously expressed across tissues (Schmid et al., 2005). Functional analyses further demonstrate that individual isoforms contribute differently to overall enzymatic activity, with certain members playing dominant catalytic roles (Clark and Lu, 2015).

Metabolic engineering strategies targeting amino acid biosynthesis have demonstrated the feasibility of improving crop nutritional quality. Expression of a feedback-insensitive AK in *Vicia narbonensis* significantly increased Met levels by 2.4-fold compared with the wild type (Demidov et al., 2003). Similarly, genetic modification of *AK* and/or dihydrodipicolinate (*DHDPS*) genes in rice enhanced lysine accumulation (Yang et al., 2016). Further qualitative investigations reveal that feedback inhibition of aspartate kinase and dihydrodipicolinate synthase plays a key role in elevating both free lysine concentrations and overall lysine accumulation in rice seeds (Yang et al., 2021). Quantitative trait locus analysis in maize identified *ZmAK2* as a regulator of multiple free amino acids, including Lys, Thr, Met, Leu, and Ile, with distinct haplotypes displaying significant phenotypic variation (Wang et al., 2007). Correlation analysis between *ZmAK2* haplotypes and the content of aspartate-derived amino acids revealed substantial genetic diversity within the *ZmAK2* locus in maize. Furthermore, the development of high-methionine maize can be accelerated through the introgression of favorable *ZmAK2* alleles into parental lines of elite hybrids using molecular breeding strategies (Duo et al., 2024).

Despite its importance, the soybean AK gene family has not been comprehensively characterized. Given the significance of soybeans in agriculture and nutrition, a detailed understanding of amino acid metabolic regulation is required. This study presents the first systematic analysis of the soybean *AK* gene family using bioinformatics approaches, including comprehensive characterization of protein physicochemical properties, sequence features, phylogenetic relationships, collinearity, promoter cis-acting elements, protein-protein interaction networks, gene expression patterns, and subcellular localization. Furthermore, transgenic soybean lines of *GmAK6* were generated, and quantitative analysis of 17 amino acids in seeds demonstrated that *GmAK6* (*Glyma.08G.107800*) may regulate the biosynthesis of aspartate-derived amino acids, particularly Met and Thr. These results provide a foundation for elucidating AK-mediated regulatory mechanisms and offer potential targets for genetic strategies aimed at enhancing essential amino acid content in soybean.

## Materials and methods

### Identification of aspartate kinase genes in soybean

Putative aspartate kinase (AK) genes in soybean were identified using both sequence similarity and domain-based screening strategies. Protein sequences of six AK genes from *Arabidopsis thaliana* were retrieved from the TAIR database (https://www.arabidopsis.org/) and used as queries for BLASTP searches against the soybean genome (Wm82.a6.v1) available in the Phytozome database (https://phytozome-next.jgi.doe.gov/). A hidden Markov model (HMM)-based search was performed using the PIRSF000726 domain profile to enhance the detection of AK family members. This combined approach identified 17 putative *GmAK* genes. Subsequent domain validation using SMART and the Conserved Domain Database (CDD) confirmed that 16 of these genes possessed intact AK domains, and these were retained for further analysis.

### Chromosomal distribution, physicochemical characterization, and phylogenetic analysis of the *GmAK* gene family

The 16 *GmAK* genes identified in soybean were designated *GmAK1* through *GmAK16* according to chromosomal order, and their genomic distribution was visualized using TBtools software. Physicochemical properties of the encoded proteins were analyzed using the ExPASy ProtParam tool (https://web.expasy.org/protparam/), including coding sequence (CDS) length, number of amino acid residues, molecular weight (MW), theoretical isoelectric point (pI), instability index, aliphatic index, and grand average of hydropathicity (GRAVY). Subcellular localization predictions were obtained using Cell-PLoc 2.0 (http://www.csbio.sjtu.edu.cn/bioinf/plant-multi/). Evolutionary relationships were assessed through multiple sequence alignment of AK proteins from soybean and *Arabidopsis thaliana* using ClustalW implemented in MEGA11 (Yuan et al., 1999). The resulting alignment was used to construct a maximum likelihood phylogenetic tree using TBtools (Tamura et al., 2021), which was subsequently visualized and annotated through the iTOL online platform for enhanced interpretation of evolutionary patterns (Letunic and Bork, 2021).

### Conserved motif and gene structure analysis of the *GmAK* gene family

Conserved motif analysis of GmAK proteins was performed using the MEME suite (https://meme-suite.org/meme/tools/meme), with parameters set to identify up to 10 motifs (Bailey et al., 2015). Gene structure analysis was conducted by aligning genomic sequences with corresponding soybean genome annotation (GFF3) files. TBtools was used to visualize and integrate conserved motifs, protein domains, and gene structural features for comparative analysis of the *GmAK* gene family (Chen et al., 2020).

### Gene duplication and synteny analysis of the *GmAK* gene family

Genome assemblies and GFF3 annotation files for *A. thaliana* and *Zea mays* (maize) were downloaded from Phytozome (https://phytozome-next.jgi.doe.gov/), whereas those for *Medicago truncatula* and *Vigna angularis* were obtained from Ensembl Plants. Gene duplication events in the soybean *GmAK* gene family were identified using MCScanX implemented in TBtools, and results were visualized using Advanced Circos (Wang et al., 2012). Comparative synteny analysis between soybean and four plant species was performed using MCScanX, and interspecies collinearity was visualized using Multiple Systeny Plot analysis.

### Protein-protein interaction network and cis-acting element analysis of *GmAK* genes in soybean

Protein-protein interaction network analysis of the *GmAK* gene family was performed using the STRING database (https://string-db.org/). Interaction datasets (string_interactions_short.tsv) were retrieved and used for network construction and visualization in RStudio. Promoter sequences (2,000 bp upstream of the start codon) of all 16 *GmAK* genes were extracted using TBtools. Putative cis-regulatory elements within these promoter regions were identified through the PlantCARE database (http://bioinformatics.psb.ugent.be/webtools/plantcare/html/), and results were visualized using TBtools (Lescot et al., 2002).

### Expression pattern analysis of *GmAK* genes

#### Meta-analysis of GmAK gene expression patterns

Transcriptome datasets from three soybean seed development stages (globular, heart, and cotyledon; PRJNA246315, PRJNA246314, PRJNA246783) were obtained from the SeedGENetwork database (http://seedgenenetwork.net/annotate). Raw sequencing reads were quality-filtered using fastp (v0.23.4) to remove adapter sequences and low-quality bases. Clean reads were aligned to the soybean reference genome (Wm82.a4.v1) using HISAT2 (v2.2.1) with default parameters. Gene expression was quantified *via* featureCounts (v2.0.3), and TPM-normalized expression profiles of *GmAK* genes were visualized as heatmaps using TBtools (v2.136).

#### Transcriptome analysis of GmAKs expression during seed development

The high-protein soybean cultivar FF1235 (46.5% protein content) and Williams 82 (39.6% protein content) were used for transcriptome analysis. Pods were collected at 5, 10, 15, and 20 days after flowering under identical growth conditions, and embryos were isolated. Three biological replicates were used per developmental stage.

Total RNA was extracted using the Plant RNA Rapid Extraction Kit (TIANGEN, Beijing, China), and RNA purity and concentration were measured using NanoDrop 2000 (Thermo Fisher Scientific, USA) and Agilent 2100/LabChip GX system (Agilent, USA). High-quality RNA samples were sequenced on an Illumina platform in PE150 mode. RNA-Seq data analysis was performed using the BMKCloud pipeline (www.biocloud.net). Gene expression levels were calculated as the FPKM metric. Differentially expressed genes were identified through KEGG annotations, and heatmaps were created with TBtools.

#### RNA extraction and quantitative RT-PCR

Total RNA was isolated from soybean tissues using TRIzol reagent. RNA integrity was evaluated by 1.5% agarose gel electrophoresis, and RNA concentration was measured using a Nanodrop ND-100 spectrophotometer. First-strand cDNA synthesis was performed using the HiScript III 1st Strand cDNA Synthesis Kit (+gDNA wiper) (Vazyme, China). qPCR reactions (10 µL total volume) contained 5 µL ChamQ Universal SYBR qPCR Master Mix, 1 µL 20-fold diluted cDNA, 0.5 µL of each primer (**Supplementary Table 1**), and 3 µL nuclease-free water. Amplification conditions consisted of initial denaturation at 95 °C for 5 min, followed by 40 cycles of 95 °C for 10 s, 54 °C for 30 s, and 65 °C for 10 s, with a final hold at 8 °C. All reactions were performed in triplicate using *GmActin* as an internal control, and relative gene expression was calculated by the 2^-ΔΔCT^ method (Pfaffl, 2001).

### Subcellular localization of *GmAK6*

The *GmAK6* coding sequence was synthesized based on the soybean reference genome (Wm82.a6.v1) (Sangon biotech, Shanghai, China) and cloned into the pMD20-T vector for sequencing confirmation. The verified sequence was inserted into the SalI site of the pRI101-GFP vector to generate a GMAK6-GFP fusion construct. Protoplasts were isolated from fully expanded rosette leaves of 4-week-old *A. thaliana* by digestion in 10 mL enzyme solution containing 1.5% (w/v) cellulase R10 and 0.75% (w/v) macerozyme R10 for 5–6 h in darkness with gentle shaking. Protoplasts were filtered through a 40 µm mesh, centrifuged at 100 × g for 3 min, and washed twice with pre-cooled W5 solution (154 mM NaCl, 125 mM CaCl_2_, 5 mM KCl, 2 mM MES, pH 5.7). Cells were resuspended in an appropriate amount of MMG solution (0.4 M mannitol, 15 mM MgCl_2_, 4 mM MES, pH 5.7) and observed under a microscope using 40×objective. For PEG-mediated transformation, 200 µL protoplast suspension was mixed with 10 µL plasmid DNA and 210 µL PEG solution (40% PEG4000, 0.2 M mannitol, 0.1 M CaCl_2_) and incubated for 30 min at room temperature. After dilution with W5 solution, protoplasts were centrifuged (100 × g, 2 min), washed twice with W5, and incubated at 28 °C in darkness for 48 h before imaging. The empty pRI101-GFP vector was used as the control, and fluorescence signals were observed using a confocal microscope.

### Overexpression of *GmAK6* in soybean

The coding sequence of *GmAK6* was amplified from the high-protein and high-methionine soybean variety FF1235 using specific primers containing SacI and XbaI restriction sites. The PCR product was digested with SacI and XbaI to produce sticky ends. The pCAMBIA3300-35S-fto vector was similarly digested with SacI and XbaI to obtain a linearized vector DNA. The digested PCR fragment was subsequently ligated into the linearized vector using T4 DNA ligase. The recombinant construct was introduced into *Agrobacterium tumefaciens* strain EHA105 and used for soybean transformation *via* the cotyledonary node method (Song et al., 2013). Putative Transgenic lines were screened by PCR and confirmed by sequencing. RNA was extracted from pods at 15 days post-fertilization to evaluate *GmAK6* expression levels. Upon maturation of the T_1_ generation, seeds from three independent overexpression lines (*OE-GmAK6-1*, *OE-GmAK6-2*, and *OE-GmAK6-3*) and the non-transgenic control (Williams 82) were collected. Seventeen hydrolyzed amino acids were quantified using HPLC (1260 Infinity I, Agilent, USA), with three biological replicates to ensure statistical reliability.

### Data processing and statistical analysis

Data applied in figures and tables were computed independently for three replicates. Data were analyzed using Microsoft Excel and SPSS software, with statistical significance determined using t-test: * p < 0.05, ** p < 0.01, *** p < 0.001. Data variability was assessed using standard deviation (STDEV). Graphical visualization was performed using GraphPad Prism, TBtools, and R software.

## Results

### Genomic identification and physicochemical characterization of *GmAK* gene family members in soybean

Genome-wide analysis identified 16 members of the *GmAK* gene family in soybean, which were designated according to their chromosomal positions (**Table 1**; **Supplementary Table 2**). The encoded proteins ranged from 176 to 916 amino acids in length, with molecular weights between 19.58 and 100.42 kDa. Theoretical isoelectric points ranged from 5.56 to 9.30, indicating variation in physicochemical properties. Protein stability assessment based on the instability index revealed that four proteins, GmAK4, GmAK7, GmAK10, and GmAK11 (25%), were predicted to be unstable (instability index > 40), whereas the remaining proteins were classified as stable. The aliphatic index varied between 70.51 and 117.27, indicating differences in thermostability among family members. GRAVY analysis showed that seven proteins (43.75%) displayed hydrophilic properties (GRAVY < 0). Subcellular localization predictions indicated that GmAK2 was localized to both the cytoplasm and chloroplasts, GmAK5 was restricted to the cytoplasm, and the remaining family members were primarily localized to chloroplasts.

**Table 1:** Physicochemical characterization of GmAK proteins in soybean.

Gene name	Length (aa)	MW (kDa)	pI	Instability index	Aliphatic index	Grand average of hydropathicity (GRAVY)	Subcellular
*GmAK1*	562	61.15	6.02	39.09	99.73	0.081	Chloroplast
*GmAK2*	338	35.59	8.3	33.64	105.56	0.159	Chloroplast; Cytoplasm
*GmAK3*	916	100.42	6.94	34.3	92.42	-0.114	Chloroplast
*GmAK4*	331	35.91	9.3	45.6	86.92	-0.045	Chloroplast
*GmAK5*	343	35.89	8.29	33.58	105.77	0.204	Cytoplasm
*GmAK6*	916	100.41	7.63	34.83	91.35	-0.125	Chloroplast
*GmAK7*	325	35.34	9.17	46.54	87.94	-0.028	Chloroplast
*GmAK8*	497	54.13	5.82	36.24	72.23	-0.411	Chloroplast
*GmAK9*	487	52.66	8.49	35.55	73.16	-0.418	Chloroplast
*GmAK10*	181	20.12	8.19	43.88	114.53	0.438	Chloroplast
*GmAK11*	176	19.58	8.89	42.83	117.27	0.466	Chloroplast
*GmAK12*	487	53.20	5.95	36.74	70.51	-0.405	Chloroplast
*GmAK13*	561	61.45	5.47	38.66	102.69	0.143	Chloroplast
*GmAK14*	565	61.59	5.96	36.7	102.12	0.107	Chloroplast
*GmAK15*	266	28.35	6.95	31.31	105.98	0.33	Chloroplast
*GmAK16*	562	61.65	5.56	39.65	102.49	0.094	Chloroplast

### Chromosomal localization and phylogenetic analysis of the *GmAK* gene family members in soybean

**Figure 1:**
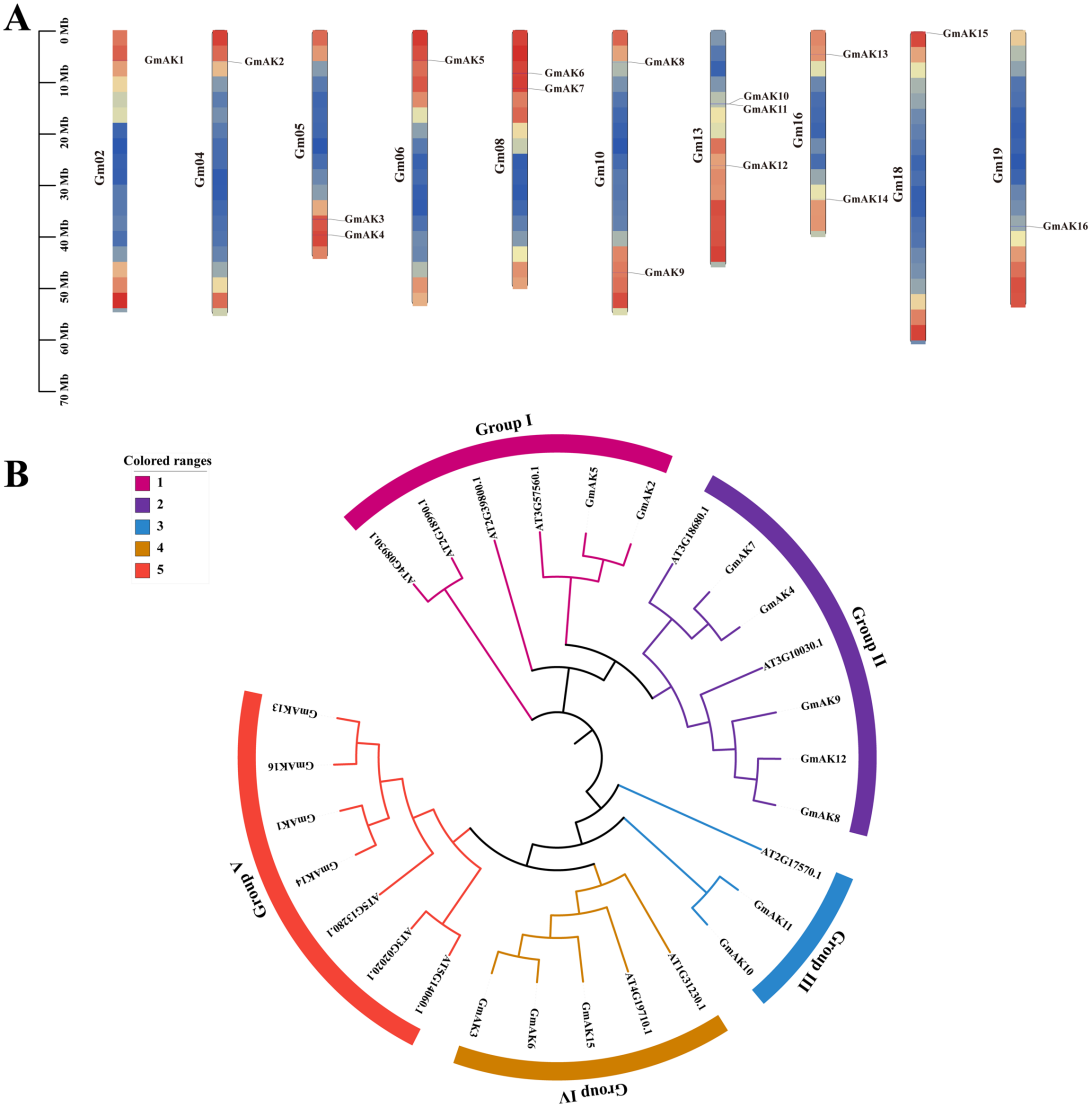
Genomic distribution and evolutionary relationships of the *GmAK* gene family in soybean. **(A)** Chromosomal localization of 16 *GmAK* genes across the soybean genome. Gene density is represented by a gradient scale (red, high density; blue, low density), and chromosome numbers are indicated on the left side of each chromosome bar. **(B)** Maximum likelihood phylogenetic tree of AK proteins from soybean and *A. thaliana*. Different evolutionary groups are distinguished by distinct colors.

The soybean *GmAK* gene family comprised 16 members, exhibiting an uneven chromosomal distribution pattern (**Figure 1A**). The highest gene concentration was observed on chromosome 13, which contained three members, including the closely linked *GmAK10*-*GmAK11* pair, indicating a probable tandem duplication event. Two genes were located on each of chromosomes 5, 8, and 16, whereas each of the remaining six chromosomes harbored a single *GmAK* gene.

Phylogenetic analysis based on full-length protein sequences from *A. thaliana* and soybean resolved the *GmAK* gene family into five major evolutionary clades (Group I-V) (**Figure 1B**). Group II represented the largest clade with five members, followed by Groups IV and V with three and four members, respectively, while Groups I and III contained two genes each, indicating evolutionary divergence within the gene family.

### Phylogenetic, conserved motif and gene structure analysis of the *GmAK* gene family in soybean

**Figure 2:**
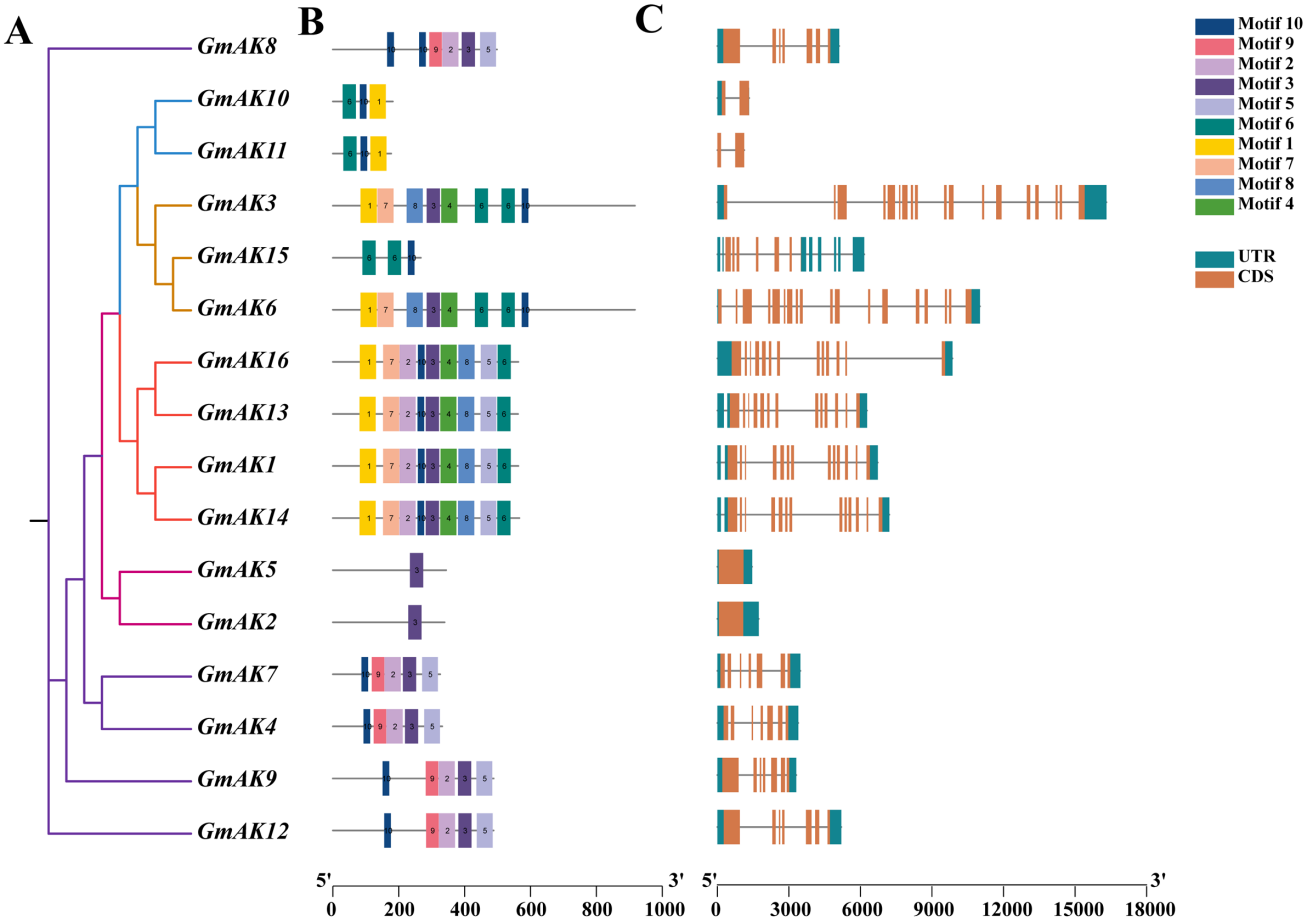
Phylogenetic tree, conserved motifs, and exon-intron structure of *GmAK* genes in soybean. **(A)** Phylogenetic tree of *GmAK* genes in soybean. **(B)** Conserved motifs of GmAK proteins. Ten motifs are color-coded for visualization. **(C)** Exon-intron structure of *GmAK* genes in soybean.

Phylogenetic analysis classified soybean *GmAK* genes into five evolutionary clades, consistent with cross-species relationships (**Figure 2A**). Conserved motif analysis showed that individual proteins contained between 1 and 9 motifs, with closely related genes displaying highly similar motif architectures. Group I members (*GmAK2* and *GmAK5*) uniquely possessed only motif3, indicating lineage-specific conservation. Group IV members (*GmAK1*, *GmAK13*, *GmAK14*, and *GmAK16*) displayed the highest structural complexity, harboring up to nine motifs. Motif10 was present in all family members except *GmAK2* and *GmAK5*, suggesting conservation across the gene family (**Figure 2B**; **Supplementary Figure 1**).

Gene structure analysis revealed variation in exon-intron organization. Most genes contained ≥ 9 exons and 3 introns (peaking in *GmAK3*/*GmAK6*), whereas *GmAK11* and *GmAK10* showed reduced exon-intron structures with 2/1 and 3/1 configurations, respectively (**Figure 2C**). This structural variability suggests extensive exon shuffling and intron loss or gain events during evolution, contributing to functional diversification within the *GmAK* family.

### Gene duplication events of the *GmAK* gene family in soybean

**Figure 3:**
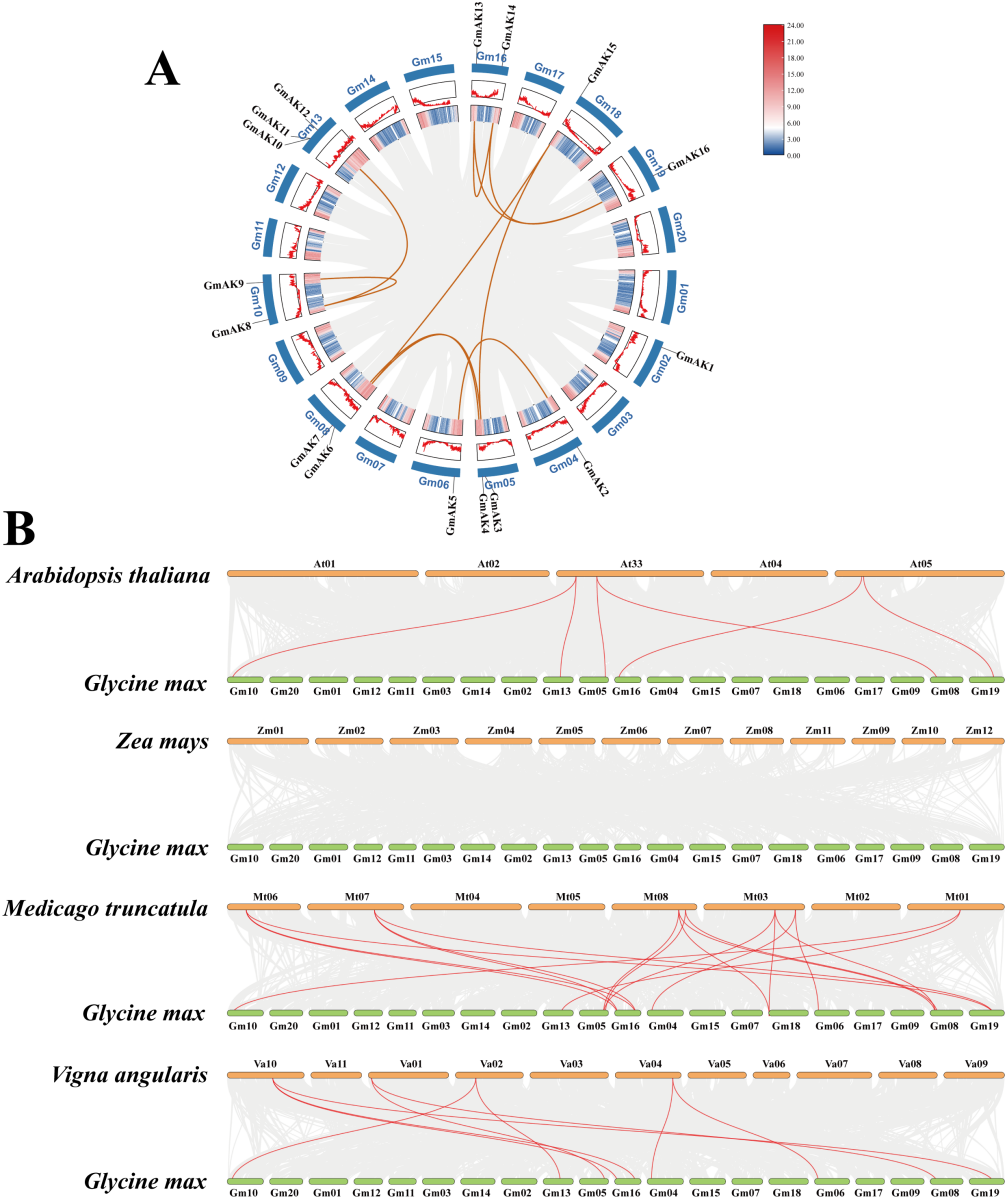
Collinearity analysis of *GmAK* genes. **(A)** Intra-genomic collinear gene pairs in soybean. **(B)** Inter-species collinearity between soybean and four representative species (*A. thaliana*, *Zea mays*, *M. truncatula*, and *V. angularis*). Grey lines indicate background synteny, while red lines highlight collinear *GmAK* gene pairs.

To explore the evolutionary mechanisms underlying the expansion of the *GmAK* gene family, 10 duplicated gene pairs were identified in soybean (**Figure 3A**). Three gene groups showing multiple duplication relationships were detected: (i) *GmAK3*, *GmAK6*, and *GmAK15*, forming three pairwise duplication connections; (ii) *GmAK13*, *GmAK14*, and *GmAK16*, generating another three pairs; and (iii) four independent duplication pairs, including *GmAK2*/*GmAK5*, *GmAK4*/*GmAK7*, *GmAK8*/*GmAK9*, and *GmAK8*/*GmAK12*. The prevalence of dispersed duplication patterns suggests that segmental and dispersed duplication events contributed to the expansion of the *GmAK* gene family.

Comparative synteny analysis across soybean, *A. thaliana*, *Zea mays*, *M. truncatula*, and *V. angularis* revealed distinct evolutionary patterns (**Figure 3B**). Six orthologous gene pairs were identified between soybean and *A. thaliana*, primarily distributed on chromosomes 3 (four pairs) and 5 (two pairs). No syntenic relationships were detected with *Zea mays*, whereas strong conservation was observed with closely related legumes, including 18 orthologous pairs with *M. truncatula* and 9 with *V. angularis*. These results indicate strong evolutionary conservation of AK genes within the Fabaceae family, with particularly high syntenic conservation between soybean and *M. truncatula*, supporting their close phylogenetic relationship.

### GmAK protein-protein interaction network and cis-acting elements in soybean promoters

**Figure 4:**
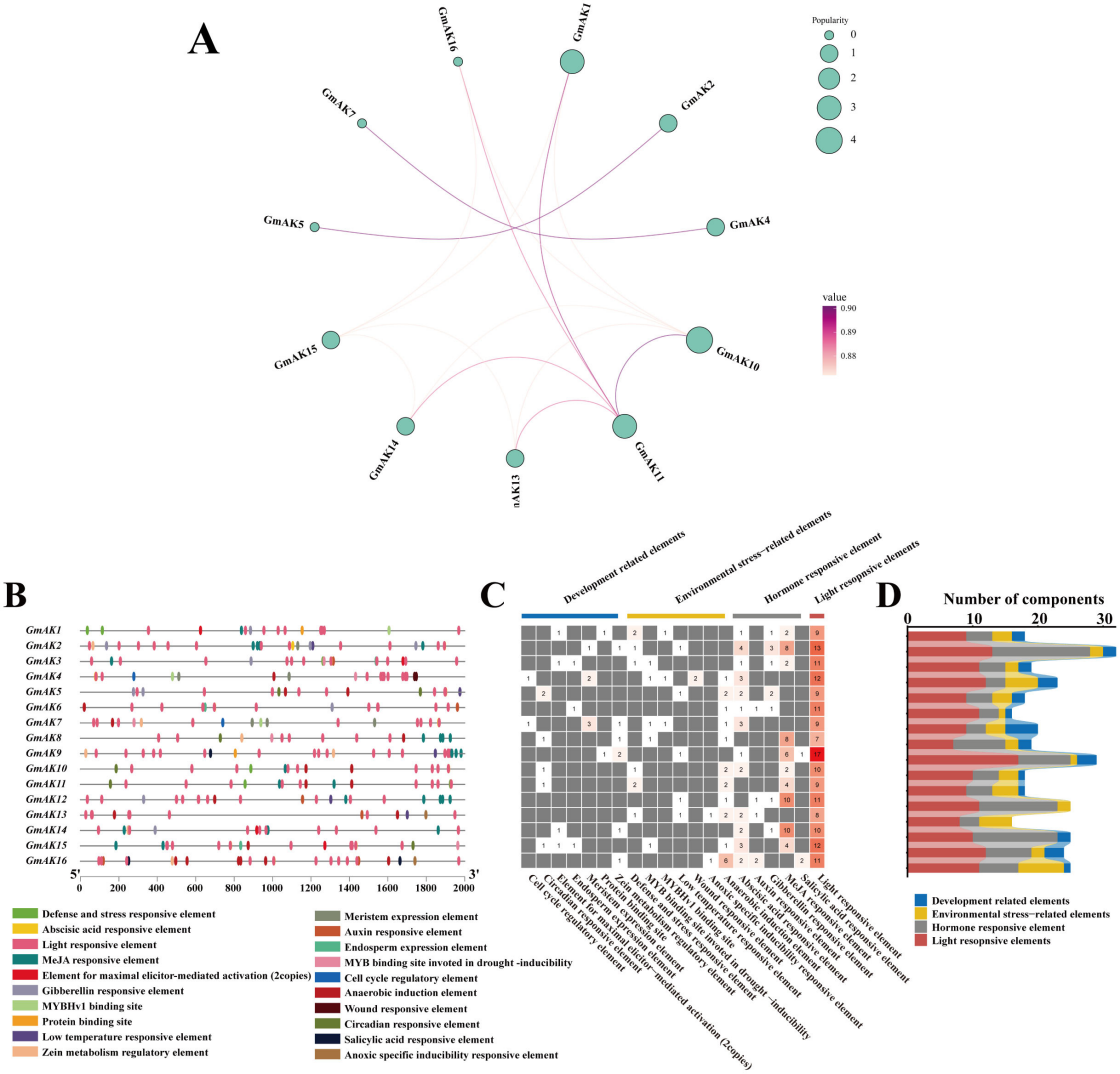
Protein-protein interaction network and cis-acting element analysis of soybean *GmAK* genes. **(A)** Predicted protein-protein interaction network of *GmAK* family members. **(B)** Distribution of cis-acting elements in soybean *GmAK* gene promoters. **(C, D)** Statistical analysis of cis-acting elements in *GmAK* gene promoters.

Protein-protein interaction analysis revealed strong connectivity among GmAK proteins, with all interactions exhibiting high confidence scores (≥ 0.8) in STRING (**Figure 4A**). The network comprised 12 interacting pairs, GmAK1, GmAK10, and GmAK11 functioned as central hub nodes within the interaction network. GmAK10 interacted with five proteins (GmAK1, GmAK11, GmAK13, GmAK14, and GmAK16), while GmAK11 also interacted with five partners (GmAK1, GmAK10, GmAK13, GmAK14, and GmAK16), suggesting their key regulatory roles.

Promoter analysis of 2 kb upstream sequences identified 345 cis-acting elements distributed across 20 functional categories (**Figure 4B**; **Supplementary Table 3**). Light-responsive elements were the most abundant (48.99%, 169/345), followed by hormone-responsive elements (29.28%, 101/345). Among hormone-related elements, jasmonic acid-responsive elements (56/101, 55.45%) and abscisic acid-responsive elements (27/101, 26.73%) were predominant, suggesting strong hormonal regulation. Additional elements associated with stress responses (e.g., low-temperature and wound response) and developmental regulation (e.g., endosperm and meristem-specific expression) indicated that *GmAK* genes are subject to complex environmental and developmental control (**Figures 4C, D**).

### Expression pattern analysis of *GmAK* genes during soybean seed development

**Figure 5:**
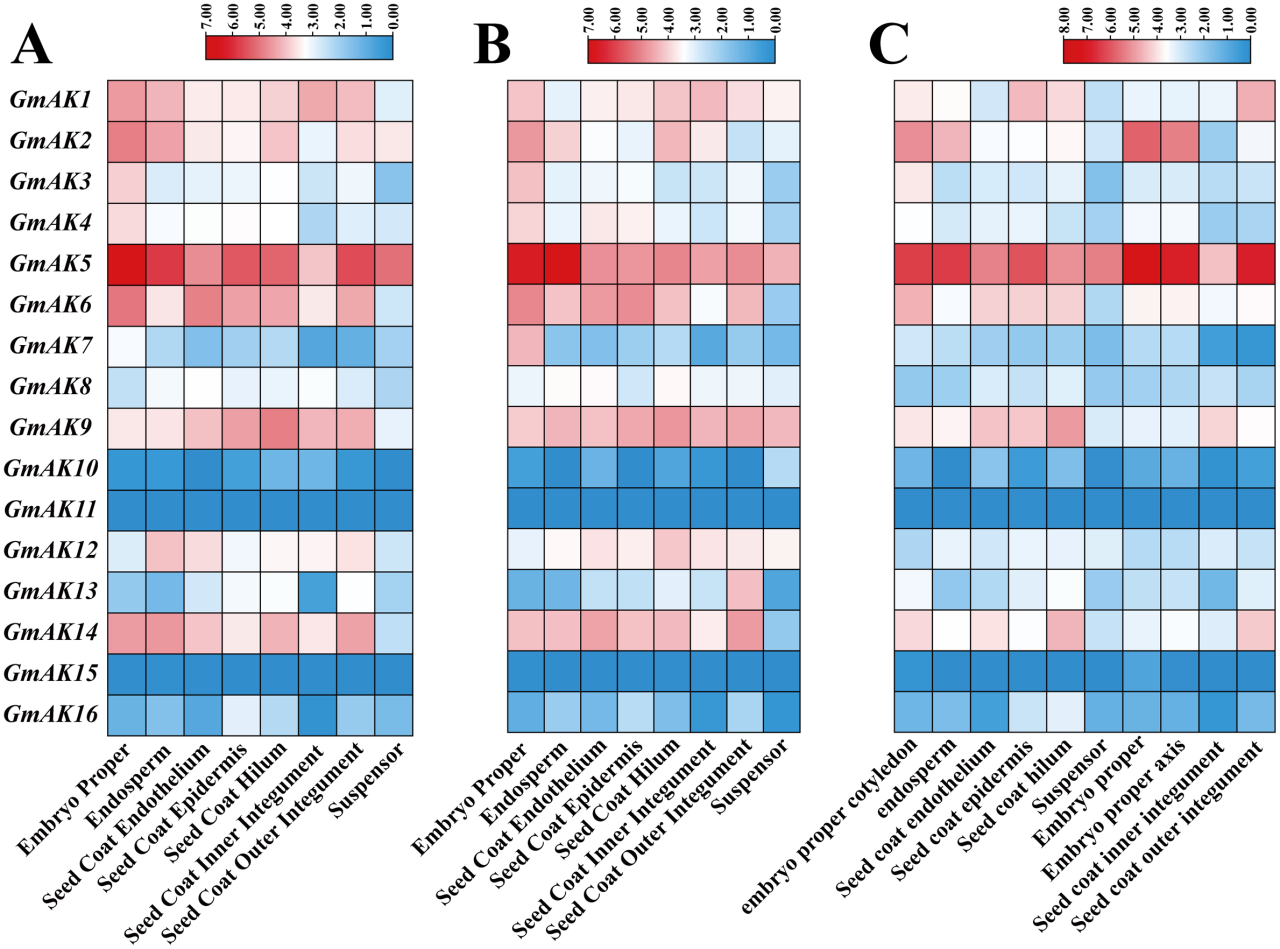
Expression profiles of soybean *GmAK* genes. **(A)** Expression patterns of *GmAK* genes during the heart stage of seed development. **(B)** Expression patterns of *GmAK* genes during the globular stage of seed development. **(C)** Expression patterns of *GmAK* genes during the cotyledonary stage of seed development.

Analysis across three key seed developmental stages (globular, heart, and cotyledonary) revealed both conserved and stage-specific expression patterns among *GmAK* family members based on publicly available transcriptome data (**Figures 5A–C**; **Supplementary Tables 4**-**6**). All 16 genes showed coordinated expression during seed development, accompanied by strong spatial and temporal regulation. *GmAK5* showed consistently high expression throughout embryogenesis. This gene displayed dynamic tissue-specific expression, with peak levels in the embryo proper and endosperm at the globular and heart stages, and in the embryo proper, embryo axis, and seed coat outer integument at the cotyledon stage. These patterns revealed stage-specific high expression of *GmAK1*, *GmAK2*, *GmAK6, GmAK9, and GmAK14* in tissues of the globular and heart-stage. These stage-specific expression patterns suggest potential roles in embryonic polarity establishment and tissue differentiation, whereas the overall expression profile of the *GmAK* gene family indicates coordinated involvement in the spatiotemporal regulation of seed development.

### Expression levels of *GmAK* genes during soybean seed development

**Figure 6:**
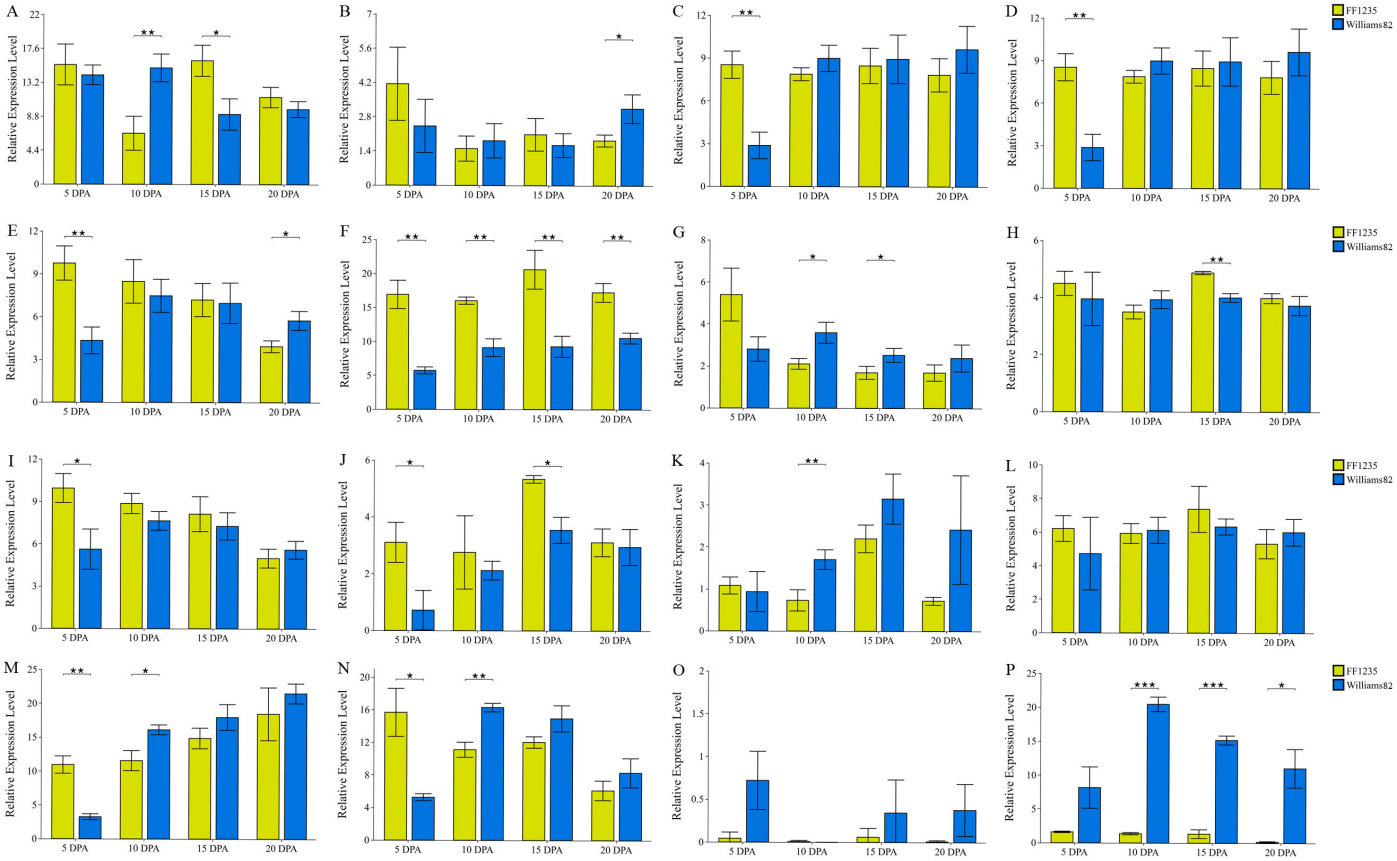
Expression levels of *GmAK* genes in soybean at different post-flowering stages based on transcriptome data. **(A-P)**. *GmAK1~ GmAK16.* Statistical significance was determined using t-test: *p < 0.05, **p < 0.01, ***p < 0.001.

Expression dynamics of *GmAK* genes were analyzed in two contrasting soybean genotypes, FF1235 and Williams 82, using transcriptome data from young pods collected at 5, 10, 15, and 20 days after flowering (DAF) (**Figures 6A–P**). Expression profiling revealed stage-specific differential expression of several *GmAK* genes between the two accessions (**Figure 6**). *GmAK6* showed statistically significant expression differences across all developmental stages, with consistently lower transcript levels in Williams 82 compared with FF1235. This divergence was most pronounced during the protein accumulation phase (15–20 DAF), suggesting a potential role of *GmAK6* in regulating amino acid biosynthesis during seed development. These results indicate that *GmAK6* may contribute to the modulation of aspartate-derived amino acid metabolism in soybean seeds.

**Figure 7:**
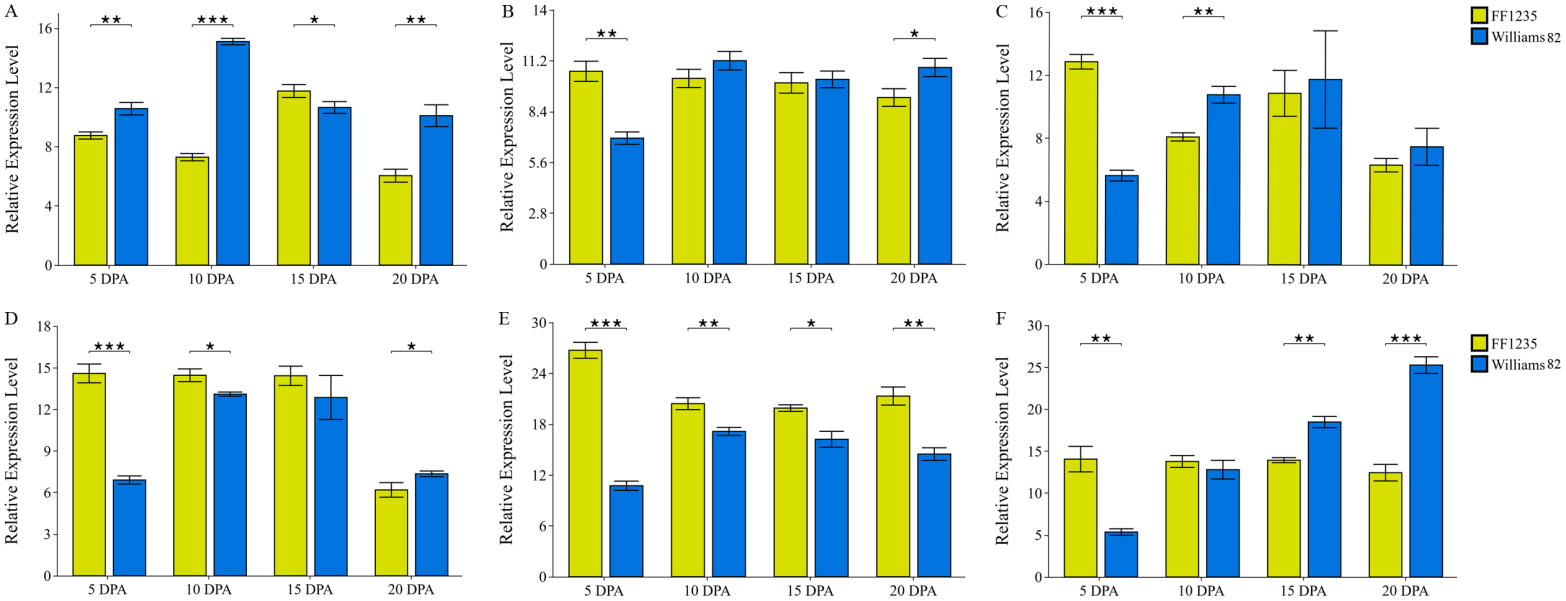
Expression levels of six *GmAK* genes in soybean at different post-flowering stages based on qRT-PCR analysis. **(A)** *GmAK1.* **(B)** *GmAK3.* **(C)** *GmAK4.* **(D)** *GmAK5.* **(E)** *GmAK6.* **(F)** *GmAK13.* Statistical significance was determined using t-test: *p < 0.05, **p < 0.01, ***p < 0.001.

To further validate the reliability of the transcriptome sequencing data, quantitative reverse transcription polymerase chain reaction (qRT-PCR) analysis was performed using RNA isolated from the same samples previously used for sequencing (**Supplementary Table 1**). Six highly expressed genes (*GmAK1*, *GmAK3*, *GmAK4*, *GmAK5*, *GmAK6*, and *GmAK13*) were selected for validation (**Figures 7A–F**). The qRT-PCR results exhibited trends consistent with transcriptome data across both genotypes, confirming the robustness of the sequencing dataset.

### Subcellular localization of *GmAK*

**Figure 8:**
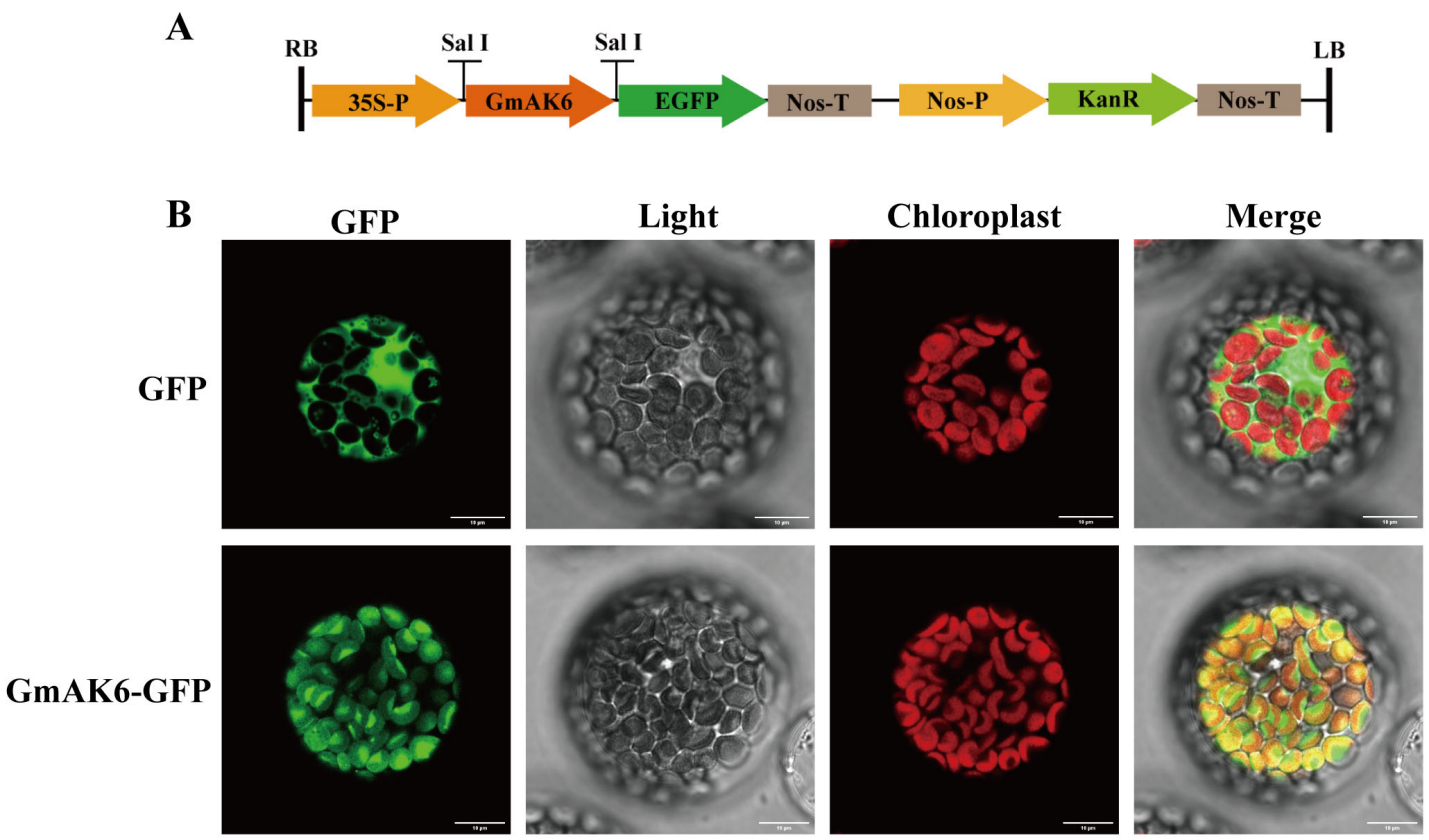
Subcellular localization of *GmAK6*. **(A)** Schematic representation of the transient expression construct. **(B)** Subcellular localization of GmAK6 protein in *A. thaliana* protoplasts.

Transient expression assays in *A. thaliana* protoplasts were performed to determine the subcellular localization of *GmAK6* using pRI101::GmAK6-GFP and pRI101::GFP as control constructs (**Figure 8A**). Co-localization analysis with chloroplast-specific markers revealed that the GmAK6-GFP fusion protein was exclusively localized to chloroplasts (**Figure 8B**), consistent with bioinformatics-based predictions (**Table 1**).

### Construction of *GmAK6* transgenic plants and their role in seed development

**Figure 9:**
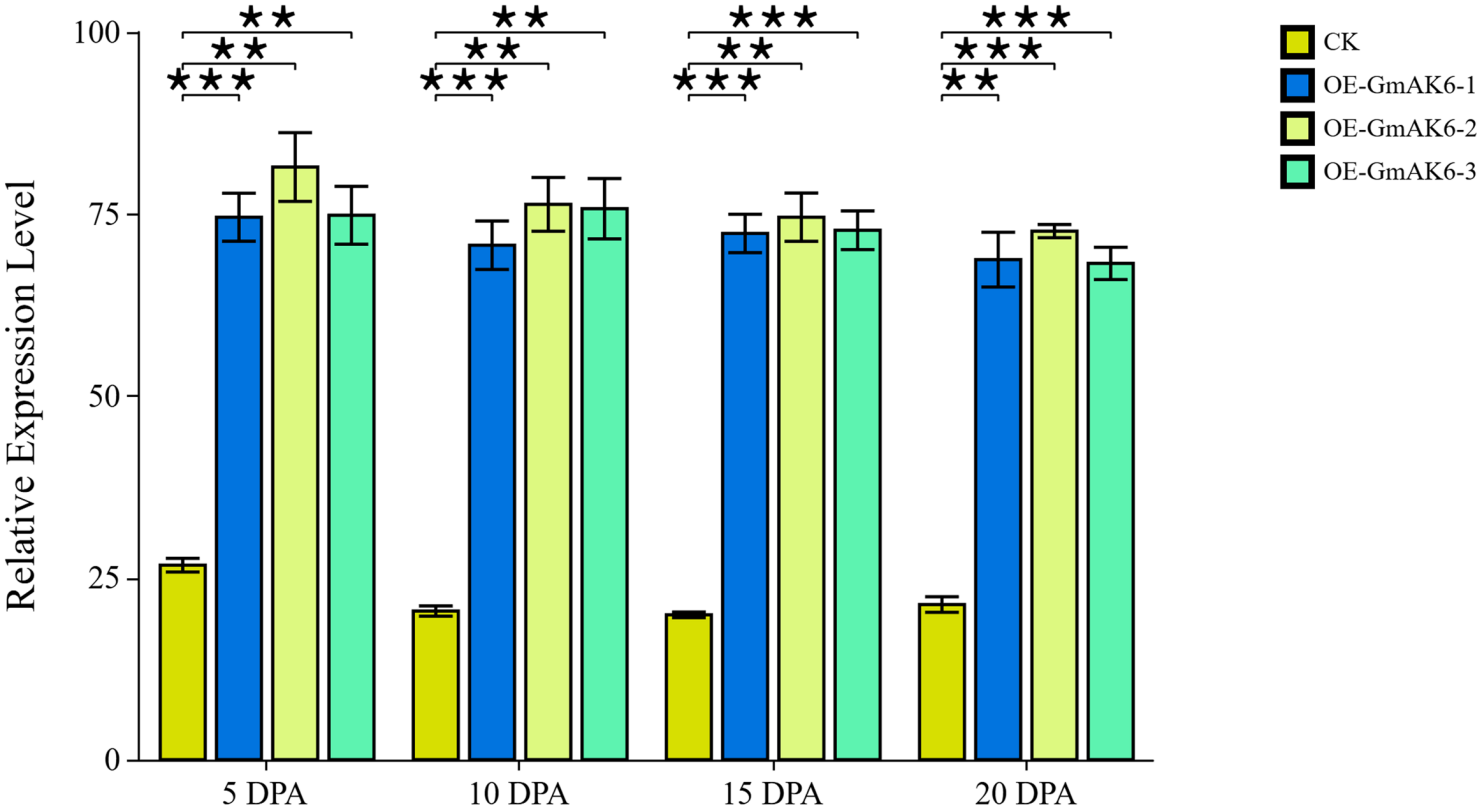
qRT-PCR analysis of three independent *GmAK6* transgenic soybean events (*OE-GmAK6-1*, *OE-GmAK6-2*, *OE-GmAK6-3*. Statistical significance was determined using t-test: **p < 0.01, ***p < 0.001.

To characterize the biological role of *GmAK6*, transgenic soybean lines were generated in the Williams 82 genetic background using a hypocotyl node transformation system (**Supplementary Figure 2**). qRT-PCR analysis of three independent transgenic events (*OE-GmAK6-1*, *OE-GmAK6-2*, *OE-GmAK6-3*) confirmed strong overexpression of *GmAK6*, with transcript levels approximately 300% higher than those in non-transgenic control plants (**Figure 9**).

**Figure 10:**
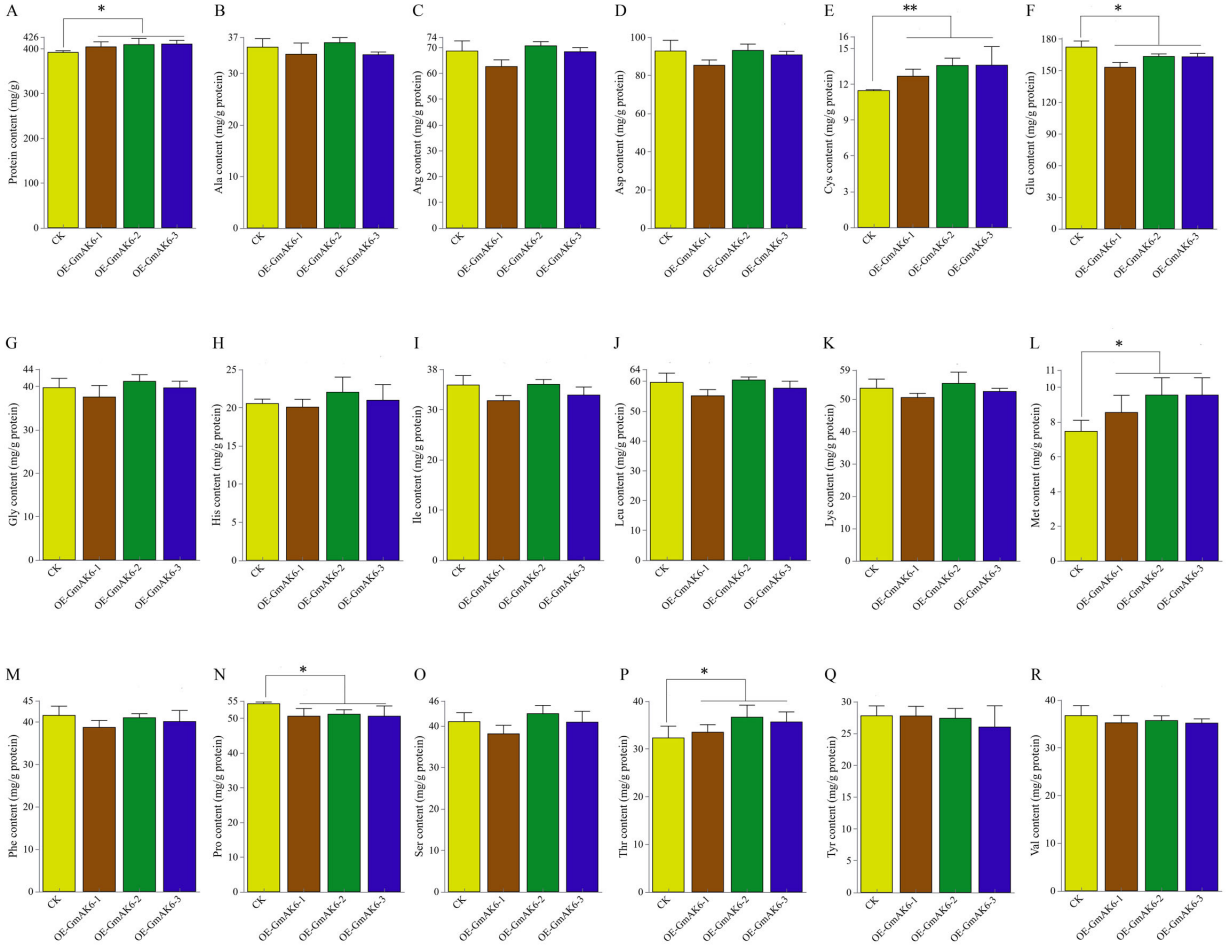
Functional Analysis of *GmAK6* in Soybean. **(A)** Total seed protein content. **(B-R)** Contents of 17 major hydrolyzed amino acids (mg/g protein). Error bars represent the standard deviation (SD) of three biological replicates (n ≥ 3). Statistical significance between overexpression lines and the control was determined using a t-test: *p < 0.05, **p < 0.01.

To evaluate metabolic changes in transgenic soybeans, total protein content and 17 hydrolyzed amino acids were quantified. Total protein content was determined by Kjeldahl nitrogen analysis, whereas amino acid composition was measured using HPLC. The results demonstrated that total seed protein content in *GmAK6* overexpression lines increased by an average of 3.70% compared with the Williams 82 control, with a statistically significant difference (**Figure 10A**). Cysteine (Cys), Met, and Thr levels were significantly increased across all transgenic lines, with Cys showing the most pronounced elevation (**Figures 10E, L, P**). As methionine and threonine are synthesized *via* the aspartate pathway, these changes are consistent with the regulatory role of AK-HSDH encoded by *GmAK6.* Cys accumulation may be indirectly influenced through methionine-associated metabolic regulation. Glutamate (Glu) and proline (Pro) contents were significantly reduced in overexpression lines (**Figures 10F, N**), whereas the remaining 13 amino acids showed no significant differences between transgenic and control plants (**Figures 10B–D, G-K, M, O, Q, R**). These results indicate that *GmAK6* contributes to the regulation of total seed protein content and plays a crucial role in modulating aspartate-derived amino acid metabolism.

## Discussion

The AK gene family has been extensively characterized in model and crop species such as Arabidopsis (*Arabidopsis thaliana*) and maize (*Zea mays*), whereas its functional and evolutionary features in soybean (*Glycine max*) remain less explored. In this study, 16 *GmAK* genes were identified and classified into five distinct phylogenetic clades. Group II contained the highest number of members, suggesting potential functional expansion and a possible lineage-specific role in soybean aspartate metabolism. However, due to the limited scope of previous studies, which have primarily focused on *Arabidopsis thaliana*, we have constructed phylogenetic trees exclusively for soybean and Arabidopsis. It is limited of using only soybean and Arabidopsis for phylogenetic analysis. Future inclusion of AK homologs from other crops is needed. Evolutionary analysis indicated that *GmAK* paralogs with close phylogenetic relationships shared similar conserved motif architectures, with motif numbers ranging from 1 to 9. This pattern is consistent with soybean whole-genome duplication events, which likely contributed to the expansion of the *GmAK* gene family. Despite high sequence similarity among duplicated genes, distinct expression profiles across tissues and developmental stages were observed, indicating functional divergence. These results suggest that structural diversification and differential regulation have contributed to the functional specialization of *GmAK* genes in soybean.

Inter-species collinearity analysis demonstrated pronounced conservation of *GmAK* genes between soybean and the legumes *M. truncatula* and *V. angularis*, consistent with their phylogenetic proximity (Takahashi and Tomooka, 2024). No syntenic relationships were detected between soybean and maize, likely reflecting substantial divergence of the AK gene family between the Leguminosae and Gramineae lineages. This divergence may also be associated with lineage-specific evolutionary pressures, including differences in amino acid composition reported in maize kernels (Huang et al., 2006).

Protein-protein interaction analysis identified *GmAK1*, *GmAK10*, and *GmAK11* as central hub genes within the regulatory network. Promoter analysis revealed a high abundance of cis-acting regulatory elements associated with growth and stress responses. These findings are consistent with previous reports indicating that modulation of amino acid biosynthetic pathways can enhance plant stress tolerance (Mishra et al., 2022).

In metabolic engineering studies, overexpression of lysine feedback-insensitive AK and DHPS has been shown to significantly enhance free lysine accumulation (Long et al., 2013), providing a promising strategy for improving soybean nutritional quality through molecular breeding. Phosphorus deficiency has been reported to modulate aspartic metabolism *via* regulation of amino acid biosynthesis pathways, highlighting the environmental responsiveness of *GmAK* genes (Mo et al., 2019; Zhu et al., 2020). Transcriptomic analysis revealed that *GmAK5* and *GmAK6* are strongly expressed during seed development, suggesting roles in amino acid biosynthesis and developmental regulation. *GmAK6* was selected for functional analysis based on its chloroplast localization, seed-enriched expression, and reported dual enzymatic activity (Ma et al., 2026; Zhou et al., 2026; Ohshida et al., 2018; Isogai and Takagi, 2021).

In transgenic plants overexpressing *GmAK6*, a significant increase in the concentrations of the aspartate-derived amino acids Met and Thr was observed, consistent with the predicted function of this gene. This result is consistent with the interpretation that *GmAK6* enhances the metabolic flux through the aspartate-derived pathway, reflecting the known role of AK as a key rate-limiting enzyme. The expression of feedback-insensitive AK in *Vicia narbonensis* has been associated with a significant increase in seed Met content (Demidov et al., 2003). In maize, different haplotypes of *ZmAK2* are significantly correlated with variations in multiple free amino acid levels (Wang et al., 2007). Our findings indicate that *GmAK6* may serve a similar “valve” function, regulating the conversion of aspartate to Asp-4-phosphate via its kinase activity and thereby modulating the metabolic flux within this pathway. Consistent with the functional differentiation of AK isoenzymes in *Arabidopsis* (Clark and Lu, 2015), the tissue-specific high expression of *GmAK6* during seed development suggests that it may be the predominant regulatory enzyme for this pathway in soybean seeds.

Overexpression of *GmAK6* results in alterations in amino acid profiles, characterized by increased levels of Met, Thr, and Cys, alongside decreased Glu and Pro. These changes may reflect the complex interactions within metabolic networks. The upregulation of the aspartate pathway may compete for common substrates or precursors, indicating a shift in nitrogen flux. Glu serves as a central molecule in plant nitrogen metabolism and a precursor for various amino acids, including Pro. Its reduced abundance suggests that nitrogen metabolic flux is preferentially directed toward aspartate and its derivatives, such as proline, to support the enhanced aspartate pathway. This phenomenon of “metabolic reallocation” is common in crop metabolic engineering, where compensatory changes in amino acid levels are observed—for example, increased lysine synthesis in rice (Yang et al., 2021). In plants, amino acid biosynthetic precursors are derived from the carbon metabolism *via* the tricarboxylic acid (TCA) cycle (Field et al., 2004; Binder et al., 2007; Galili et al., 2016). Glutamic acid is synthesized from α-ketoglutarate and serves as a key precursor for proline biosynthesis (Stein et al., 2011). Aspartate biosynthesis depends on oxaloacetate and provides the metabolic entry point for the synthesis of methionine, threonine, and isoleucine (Jander and Joshi, 2010). Moreover, aspartate kinase (AK) activity is subject to synergistic feedback inhibition by lysine and threonine, which play a central role in regulating flux through the aspartate pathway (Stephane et al., 2003; Petit et al., 2018). In this study, enhanced AK-associated metabolic activity was accompanied by reduced accumulation of amino acids associated with Glutamic acid metabolic pathway. These results suggest strong interconnections among amino acid biosynthetic pathways in plants. Alteration of a key metabolic enzyme can perturb pathway-specific flux and, consequently, reshape amino acid composition across multiple biosynthetic branches. Future studies should include time-course amino acid profiling during seed development, *GmAK6* mutants construction and *in vitro* AK enzyme activity assays to further elucidate the functional role of *GmAK6* in seed amino acid metabolism. The underlying regulatory mechanisms are likely complex and warrant further detailed investigation.

## Conclusion

This study provides the first systematic identification and comprehensive characterization of the aspartate kinase (AK) gene family in *Glycine max* (soybean), substantially advancing current understanding of its functional roles in this species. A total of 16 *GmAK* genes were identified and found to be unevenly distributed across 10 chromosomes, and phylogenetic analysis grouped them into five distinct clades, clarifying their evolutionary relationships. Evolutionary evidence indicated that segmental duplication was the primary mechanism driving expansion of the *GmAK* gene family. Conserved motif and gene structure analyses further revealed that paralogous genes sharing common ancestry generally retain similar architectures, although observable variations suggest functional diversification during evolution. Promoter analysis demonstrated that nearly half of the cis-acting elements are associated with phytohormone signaling and abiotic stress responses, indicating that *GmAK* genes are likely involved in regulating growth, development, and environmental adaptation. Expression profiling during seed development revealed distinct spatial and temporal expression patterns among *GmAK* family members. *GmAK5* and *GmAK6* exhibited high expression levels, suggesting important roles in amino acid metabolism and seed development. Subcellular localization analysis further confirmed that GmAK6 was localized in chloroplasts. Functional characterization demonstrated that overexpression of *GmAK6* significantly enhanced amino acid metabolism, as transgenic lines showed increased seed protein content and elevated levels of methionine, threonine, and Cys, accompanied by reduced Glu and Pro contents. These results indicate that *GmAK6* modulates the biosynthesis of aspartate-family amino acids (Met and Thr) and influences the levels of Cys, Glu, and Pro, suggesting broader metabolic crosstalk.

## Data Availability

The raw sequencing data generated in this study have been deposited at the Genome Sequence Archive (GSA) at the National Genomics Data Center, accession number CRA076767.
