# Supplementary material for: Genome-wide identification of the Glycine max (soybean) aspartate kinase (AK) gene family and functional analysis of the GmAK6 gene in seed amino acid metabolism
Source: Front Plant Sci. 2026 Aug 4;17:1868742. doi: 10.3389/fpls.2026.1868742 (PMC13481432; doi:10.3389/fpls.2026.1868742)
Supplement: Supplementary file 1 [file DataSheet1.docx]

**Genome-wide identification of the *Glycine max* (soybean) aspartate kinase (AK) gene family and functional analysis of the *GmAK6* gene in** **seed amino acid metabolism**

**Figure S1.** Sequence logos of conserved motifs.

**Figure S2.** Agrobacterium-mediated genetic transformation of soybean cotyledon nodes. (A) Schematic representation of plant expression vector. (B) Co-culture. (C) Recovery culture. (D) Induce budding. (E) Induce bud elongation. (F) Induce rooting. (G) Sequencing (Partial data).
